# “What Bugs Me”: children’s lived experience of asthma

**DOI:** 10.1186/1710-1492-8-S1-A1

**Published:** 2012-11-02

**Authors:** Shauna Filuk, Cathy Gillespie, Bev Kulbaba, Nancy Ross, Lesley Stewart, Jo-Anne St-Vincent

**Affiliations:** 1 The Children’s Asthma Education Centre, Winnipeg, Manitoba, Canada

## Introduction

Children learn to adapt to asthma symptoms. Usual questions focused on clinical signs (eg., have you wheezed or used your blue puffer today?) can lead to the assumption that asthma is well controlled and has little impact on daily life. More focused queries can lead to better information about asthma control. Parents may not be aware of the impact of asthma on their child.

## Objectives

Educators at the Children’s Asthma Education Centre sought ways to help children express the impact of asthma on their daily life, and to improve insight into children’s lived experience of asthma.

## Methods

The “What Bugs Me” questionnaire was developed using lived experiences frequently expressed by children with asthma. We began a pilot study with children age 7-11 years attending a Family Asthma Program and subsequently studied both children and parents. Parents and children completed the questionnaire separately and shared their findings at the end of the session.

## Results

**Table 1:** 

Issues of concern:	Children n=65	Parents n=25
* Coughing at night	39/65 (**60%**)	21/25 (**84%**)
Trouble breathing during gym	37/65 (57%)	14/25 (56%)
Can’t run	34/65 (52%)	7/25 (28%)
* Worried about dying	29/65 (**45%**)	4/25 (**16%**)
Coughing with laugh	22/65 (32%)	4/25 (16%)
Using puffers in public	22/65 (34%)	5/25 (52%)
Can’t have the pet I want	22/65 (34%)	11/25 (44%)
Missing activities when sick	22/65 (34%)	8/25 (32%)

Surprisingly, 45% of children but only 16% of parents noted they worry about dying (p = 0.014). Fewer children (60% vs 84% parents, p = 0.045) noted nocturnal cough as a problem. More children (52% vs 28% parents, p = 0.058) noted they “couldn’t run”.

## Conclusion

There were significant disconnects between children and their parents. Asthma Educators and clinicians should direct questions related to the lived experience of asthma to the child. Focused questions can help parents and educators gain insight into the impact of asthma of the child’s social and emotional well being in order to address issues of importance.

